# Supplementary material for: A Review on the Current Treatment Paradigm in High-Risk Prostate Cancer
Source: Cancers (Basel). 2021 Aug 24;13(17):4257. doi: 10.3390/cancers13174257 (PMC8428221; doi:10.3390/cancers13174257)
Supplement: Supplementary file 1 [file cancers-13-04257-s001.zip › cancers-1338594-supplementary.pdf]

Treatment Modality	Advantages	Disadvantages	Evidence	
**Radiotherapy**			Studies	Outcomes
Dose Escalation				
Brachytherapy Boost	Long-term durable improvement in biochemical control vs. EBRT boost<br>No change in GU morbidity with HDR boost	No overall survival benefit vs. EBRT boost<br>Increased acute and late GU morbidity with LDR boost<br>Trend towards increased GI morbidity	ASCENDE-RT: dose-escalation with LDR brachytherapy boost vs. EBRT boost	15-year biochemical progression-free survival 80% with brachytherapy boost vs. 53% with EBRT boost<br>GU morbidity higher in LDR brachytherapy arm
Focal Lesion Boost	Improved biochemical control vs. no boost<br>No increased GI or GU toxicity	No benefit to prostate cancer-specific survival or overall survival apparent too date<br>Dominant intraprostatic lesion delineation requires expertise	FLAME: mpMRI-guided focal boost to macroscopically visible tumor vs. no boost	5-year biochemical disease-free survival improved with focal boost (HR: 0.45)
SBRT	Patient convenience<br>Resource allocation	Mild increased GU toxicity	HYPO-PROST: 15Gy/2 boost vs. Conventional fractionation after 46Gy to pelvis<br><br>HYPO-RT-PC: ultra-hypofractionation vs. conventional fractionation	No difference in 5-year biochemical-free survival, metastasis-free survival or overall survival<br><br>No difference in 5-year failure-free survival<br>Grade 2 or worse GU toxicity 6% vs. 2%
Elective Nodal irradiation	Two studies that demonstrate improved biochemical control	Longer follow-up is required<br>True value yet to be clarified but awaiting RTOG 0924	RTOG 0534: prostate bed RT alone vs. addition of ADT vs. ADT + elective nodal EBRT	5-year biochemical progression-free survival 71%, 83% and 89%, respectively

			POP-RT: whole pelvis EBRT vs. prostate only EBRT in high-risk N0 patients	Whole pelvis EBRT improved 5-year biochemical progression-free survival (HR: 0.23), 5-year disease-free survival (HR: 0.40) and 5-year metastasis-free survival (HR: 0.35), but did not impact overall survival
**Systemic Therapy**				
ADT				
Adjuvant (concurrent and following RT)	Improved time until progression and reduced incidence of metastases vs. neoadjuvant		Meta-analysis of OTT-0101 and RTOG 9413: adjuvant vs. neoadjuvant ADT	Reduced risk of metastasis with adjuvant ADT (HR: 1.40) and absolute difference of 10.8 months in mean survival time for progression
Docetaxel	Improved 4-year overall survival when combined with RT + ADT (in RTOG 0521)<br>Improved relapse-free survival (in GETUG-12)<br><br>Improved failure-free survival (in STAMPEDE)	Adverse events doubled with addition of docetaxel (in RTOG 0521)<br>No difference in metastasis-free survival or cancer-specific survival (in GETUG-12)<br>No benefit to overall survival (in STAMPEDE)	RTOG 0521: RT + ADT + docetaxel vs. RT + ADT<br><br>GETUG-12: ADT + docetaxel + extramustine vs. ADT	4-year overall survival 93% vs. 89%<br><br>Significantly improved 12-year relapse-free survival (HR 0.75), but no difference in metastasis-free survival or cancer-specific survival

			STAMPEDE: standard of care + docetaxel vs. standard of care	Improved failure-free survival (HR 0.70), but no overall survival benefit
Abiraterone	Improved failure-free survival and overall survival vs. ADT		STAMPEDE: ADT vs. ADT + abiraterone + prednisone	Improved failure-free survival (HR: 0.21) and overall survival (HR: 0.61)
Immunotherapy	Evidence of benefit in metastatic castrate-resistant setting	No level 1 evidence of benefit in high-risk setting	KEYNOTE-1999: metastatic castrate-resistant prostate cancer treated with pembrolizumab	Disease control varied with PD-L1 expression and varied between 9% and 22%
PARP inhibitors	Identifying more genes that are important in biological aggressiveness of prostate cancer that can potentially be targeted<br>Mounting evidence of utility in metastatic castrate-resistant setting	No level 1 evidence of benefit in high-risk setting	PROfound: metastatic castrate-resistant prostate cancer with alteration in BRCA1, BRCA2 or ATM or alterations in 12 other DNA damage repair genes treated with olaparib or enzalutamide/abiraterone	Improved progression-free survival (HR: 0.34) with median overall survival of 18.5 months in cohort A and 15.1 months in cohort B
**Surgery**	Some retrospective reviews suggest similar outcomes with RT<br>Greater use of MRI and PET may select patients appropriate for surgery	No randomized data comparing surgery and RT in high-risk patients	No prospective data	

Table S1. The advantages and disadvantages of various treatment options in the management of patients with high-risk prostate cancer.
